# Genome-Wide Identification and Expression Assessment for the *Phosphate Transporter 2* Gene Family Within Sweet Potato Under Phosphorus Deficiency Stress

**DOI:** 10.3390/ijms26062681

**Published:** 2025-03-17

**Authors:** Hongyang Li, Cici Bao, Huixian Xing, Xin Guo, Shujuan Wang, Xianming Zhou, Yanhui Lin, Chengcheng Si

**Affiliations:** 1 School of Tropical Agriculture and Forestry, Sanya Institute of Breeding and Multiplication, Hainan University, Haikou 571100, China; 2 Institute of Food Crops, Hainan Academy of Agricultural Sciences/Hainan Key Laboratory of Crop Genetics and Breeding, Haikou 571100, China

**Keywords:** sweet potato, phosphorus deficiency stress, *IbPHT2*

## Abstract

Hainan’s unique climate significantly contributes to soil acidification, causing phosphorus fixation into insoluble compounds, leading to phosphorus deficiency and reduced yield in sweet potatoes. The *Phosphate Transporter 2 (PHT2)* family, a group of trans-membrane phosphate transporters, is crucial for phosphate transport, distribution, and homeostasis regulation. Two *PHT2* genes, *IbPHT2-1* and *IbPHT2-2*, were first identified in sweet potato, and a phylogenetic analysis of 46 species showed high conservation of the *IbPHT2* gene family throughout plant evolution. Tissue-specific expression patterns of *IbPHT2* genes were determined in four sweet potato varieties using transcriptome analysis and RT-qPCR. The results demonstrated that *IbPHT2* was predominantly expressed in shoots, mature leaves, stems, and fibrous roots. Under phosphorus deficiency stress, *IbPHT2-2* expression was upregulated in shoots, mature leaves, and fibrous roots, with higher expression in mature leaves compared to *IbPHT2-1*. This observation suggests that, in the context of phosphorus deficiency stress, *IbPHT2-2* assumes a more pivotal function in the response mechanism. The expression levels of *IbPHT2-2* presented a negative relationship with fresh leaf weight (FLW) as well as fibrous root number per plant (FRNPP) and fibrous root weight per plant (FRWPP) based on correlation analysis. The restrictive function of *IbPHT2-2* became impaired by phosphorus deficiency, which resulted in inhibited leaf and root development of sweet potato. The findings of this study provide preliminary evidence that *IbPHT2-2* is a key gene involved in the response to phosphorus deficiency stress, influencing phosphorus absorption and distribution in sweet potato. This research contributes to our understanding of the molecular mechanisms underlying phosphorus utilization in sweet potato and may inform future strategies for improving phosphorus use efficiency in this important crop.

## 1. Introduction

Sweet potato (*Ipomoea batatas* L.) is a globally significant staple and cash crop, known for its high yield, stability, adaptability, and nutritional value, with applications in food, feed, and industry [1]. Hainan Island, situated in southern China, has emerged as the nation’s sole “tropical agricultural production base” owing to its unique climatic conditions. However, the island faces severe soil degradation stemming from forest land reclamation and soil erosion [2]. The available phosphorus content in farmland across many areas of Hainan Province reached its lowest value (11.27 mg kg^−1^) in 2015. In Changjiang, Hainan, for instance, 90% of the soil exhibits a pH between 4.6 and 6.6, characteristic of typical acidic soil [3,4]. Farmers rely heavily on large quantities of chemical fertilizers to sustain crop yields. Statistical data reveal that the average phosphate fertilizer application in Hainan Province surpasses the average phosphate fertilizer application in mainland China by 66% (59 kg P ha^−1^ in mainland China, 98 kg P ha^−1^ in Hainan Island) [5]. The substantial input of phosphate fertilizer, combined with the high temperature and humidity of the climatic environment, leads to the rapid fixation of orthophosphate (H_2_PO_4_^−^/HPO_4_^2−^) in the soil into insoluble phosphate compounds by Fe^3+^ and Al^3+^. The release of H^+^ by metal ions contributes to soil acidification [6]. Over time, these insoluble phosphate compounds constitute an increasingly higher proportion of total phosphorus, resulting in progressively more prominent phosphorus deficiency in crops and shaping the acidic red soil belt of Hainan [7,8]. Numerous studies have demonstrated Inorganic Phosphate (Pi) essential role in sweet potato growth. Phosphorus deficiency impedes sweet potato development, causing leaf yellowing, stunted growth, poor root development, and reduced photosynthetic rate, negatively impacting biomass accumulation [9,10]. Prolonged phosphorus deficiency can hinder the overall development of sweet potatoes, ultimately compromising both yield and quality [11].

Phosphate is absorbed by root cells, translocated via the xylem to the stem, and allocated to various tissues and organelles for growth and development. Superfluous phosphate is sequestered within vacuoles [12]. The function of Pi within organelles, such as the plasma membrane and chloroplasts, is crucial for the normal operation of these structures. The *PHT2* phosphate transporter in chloroplasts is responsible for Pi accumulation in leaves and mediates Pi distribution throughout the plant, regulated by transcription factors [13,14]. Furthermore, Pi homeostasis in chloroplasts is regulated by three Pi transporters localized to the chloroplast thylakoid membrane or inner membrane. These include *PHT2*, some *PHT4* members, and the plastid phosphate transporter, with *PHT2* playing a central role [15].

Numerous investigations have been conducted on the *PHT2* gene family across a wide range of plant species, such as *Arabidopsis* [16], rice [17], potato [18], and tomato [19]. Most species possess a single *PHT2* gene, whereas soybean [20] and poplar [21] have been found to harbor two gene members. Subcellular localization studies utilizing GFP fusion proteins have revealed that *PHT2* family members are predominantly localized to plastid membranes, albeit with species-specific variations. In *Arabidopsis* and wheat, *PHT2* proteins are found in the inner membrane of plastids [22,23], while in rice, *OsPHT2;1* is localized to the chloroplast envelope [17]. Potato *PHT2* has been observed in plastids [24] These findings underscore the diversity in subcellular localization of phosphorus transporters across different plant species. Plastids can be categorized into non-photosynthetic (white bodies, colored bodies) and photosynthetic (chloroplasts) types based on their photosynthetic capabilities [25]. In *Arabidopsis*, *PHT2*;1 exhibits high expression in leaves and plays a crucial role in phosphorus transport within plastids. The *atPHT2;1* mutant demonstrates impaired redistribution of Pi between young and old leaves [13]. Similarly, under phosphorus deficiency, the rice *OsPHT2;1* mutant maintains unchanged Pi concentrations in roots while experiencing a significant decrease in leaf Pi levels. Furthermore, a marked reduction in 32P radioisotope accumulation in the above-ground parts of the mutant was observed, accompanied by a decrease in photosynthetic rate compared to the wild type. These observations highlight the critical role of *OsPHT2;1* in photosynthesis [17]. In soybean, both *PHT2* gene members exhibit increased expression in leaves under phosphorus deficiency, with *GmPHT2;2* showing significantly higher upregulation compared to *GmPHT2;1*. The wheat *TaPHT2;1* mutant displays reduced Pi concentrations in chloroplasts and, irrespective of phosphorus availability, exhibits stunted growth and diminished photosynthetic capacity [26]. Conversely, the expression profile of poplar *PHT2* genes reveals that *PtPHT2.1* and *PtPHT2.2* are predominantly expressed in roots, with *PtPHT2.2* showing a significant increase in expression under low phosphorus stress [27]. Despite the extensive research conducted on *PHT2* phosphorus transporters in numerous plant species, a significant lacuna persists in our comprehension of the genome-wide characteristics and functions of *PHT2* transporters in sweet potatoes.

In this study, two members of the *PHT2* gene family, designated as *IbPHT2-1* and *IbPHT2-2*, were identified in sweet potato for the first time. A comprehensive analysis was conducted on various aspects of *IbPHT2*, including gene structure, conserved motifs, conserved domains, protein structure, phylogenetic relationships, promoter characteristics, phosphorylation sites, and trans-membrane domains. RNA sequencing (RNA-seq) was employed to analyze gene expression across various tissues, while RT-qPCR was utilized to confirm the primary expression sites. Additionally, RT-qPCR assessment was conducted to investigate the response of *IbPHT2* genes to phosphorus deficiency stress. The findings of this study provide valuable candidate genes for the future development of high-yield and high-quality sweet potato varieties through genetic engineering. These results are of significant importance for enhancing phosphorus utilization efficiency and stress resistance in sweet potatoes, offering potential for genetic improvement in the crop.

## 2. Results

### 2.1. Characterization and Examination of the PHT2 Family Genes

**Figure 1:**
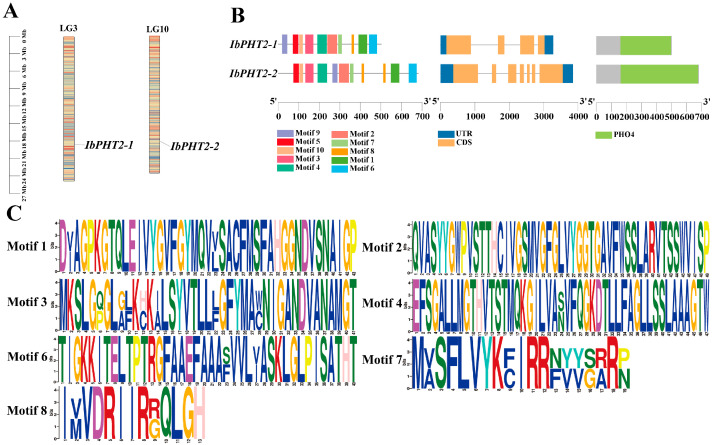
Chromosomal localization and structural assessment for the *PHT2* gene. (**A**) The chromosomal positions for *IbPHT2* genes in sweet potato are illustrated. Each basic unit represents a chromosome length of 3.0 Mb. (**B**) From left to right, the conserved motifs, gene structures, and conserved domains of the sweet potato *PHT2* gene family are depicted. The distribution of conserved motifs in *IbPHT2* is represented by colored boxes for motifs 1–10, with a scale of 100 amino acids. The genetic structure of *IbPHT2* genes comprises exons (depicted as yellow rectangles), as well as untranslated regions (UTRs, blue rectangles), with a scale of 1kb. The conserved structural domain of *IbPHT2* genes is depicted as a single green conserved structure, with a scale of 100 amino acids. (**C**) Visualization of the conserved motif of *IbPHT2* proteins in sweet potato is presented.

Two *PHT2* gene family members were identified in sweet potato genomic data, mapped to chromosomes LG3 and LG10, and named *IbPHT2-1* and *IbPHT2-2* (Figure 1A). Sequence analysis revealed that the *IbPHT2-1* and *IbPHT2-2* proteins comprised 502 and 682 amino acid residues, respectively. The molecular weights of *IbPHT2-1* and *IbPHT2-2* were determined to be 52.30 kDa and 72.98 kDa. The isoelectric points of both proteins were found to be 9.13 and 9.52, indicating their stability, as these values exceeded 7. Furthermore, the instability coefficients for *IbPHT2-1* and *IbPHT2-2* were calculated as 25.44 and 35.26, respectively, both below the threshold of 40, suggesting relatively stable protein structures. The mean hydrophilicity values were determined to be 0.521 for *IbPHT2-1* and 0.305 for *IbPHT2-2*, both positive, indicating the hydrophobic nature of these proteins. According to the predicted results, both members of the *IbPHT2* gene family were localized to the chloroplasts (Table 1). The conserved motifs of the *PHT2* proteins were predicted employing the MEME tool. NCBI-CDD analysis revealed that both members of the *PHT2* family contained conserved motifs (motifs 1–10). The conserved motifs shared among members of the same gene family were found to be highly similar. The amino acid initiation sites for the conserved domains were identified at positions 161 and 162, with domain lengths of 337 and 516 amino acids, respectively. Moreover, the members of the *IbPHT2* gene family were found to contain 3–6 introns and 4–7 exons (Figure 1B). Since motif1-8 was contained within the preserved region of the two constituents of the *PHT2* gene family, this conserved motif was visualized (Figure 1C).

### 2.2. Predictive Analysis of PHT2 Protein Structure and Interaction Network in Sweet Potato

**Figure 2:**
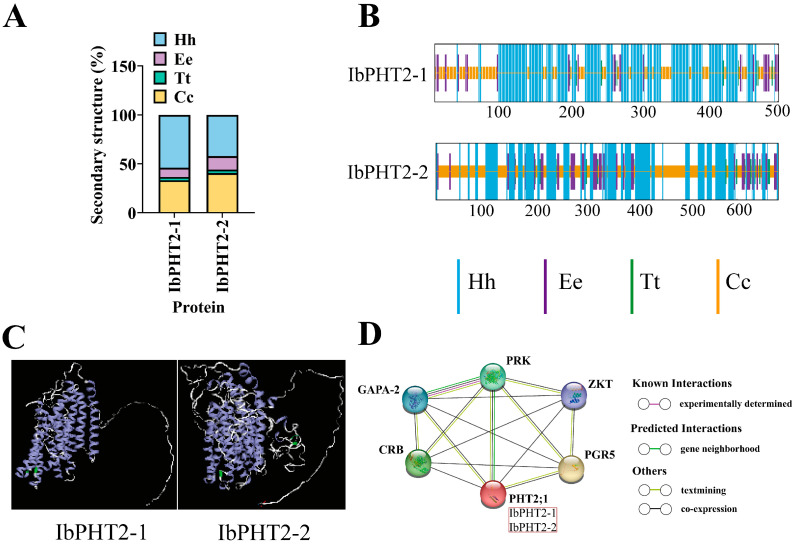
Prediction of the protein structure of sweet potato *PHT2* and protein interaction network. (**A**,**B**) Illustrates the predicted secondary structure of the *IbPHT2* protein. The blue Hh denotes α-helix, purple Ee indicates extended strand, green Tt represents β-turn, and yellow Cc signifies random coil. (**C**) Homology modeling and trans-membrane helix prediction. The tertiary structure of the sweet potato *PHT2* gene protein was predicted employing homology modeling. All proteins depicted in the figure exhibit multiple helix structures (blue regions), with the N-terminal and C-terminal portions (orange regions) primarily composed of irregular coils. (**D**) Network prediction of sweet potato *PHT2* protein interactions.

*PHT2* proteins exhibited consistent composition and structure, mainly comprising α-helices, extended strands, β-turns, and random coils. Notably, *IbPHT2-1* exhibited 87.25% α-helix and random coil content, while *IbPHT2-2* displayed 82.7% of these components. These findings indicate that α-helices and random coils constitute the primary elements of the *PHT2* protein secondary structure (Figure 2A,B, Table S1).

Tertiary structure models of homologous proteins were constructed employing homology modeling and trans-membrane helix prediction via an online tool (Figure 2C). The optimal model for *IbPHT2-1* demonstrated 74% similarity to its template, whereas *IbPHT2-2* exhibited 70% similarity. These predictions aligned with anticipated results. Both *IbPHT2* proteins displayed multiple helical structures, suggesting the presence of several trans-membrane helices, likely corresponding to the trans-membrane regions of *PHT2* phosphorus transporters. The channel structure formed by these trans-membrane helices plays a crucial role in phosphorus transport, facilitating the efficient passage of phosphorus ions across organelle membranes. The α-helices contribute to protein stability and functionality, indicating high conservation of the *PHT2* protein trans-membrane region. Random coil regions may facilitate interactions with other proteins, supporting the transport function.

Analysis of the *PHT2* protein interaction network in sweet potato (Figure 2D) revealed connections between *PHT2* and proteins of unknown function. The network exhibited a mean local clustering coefficient of 1, as well as a protein–protein interaction enrichment *p*-value of 2.03 × 10^−3^. Results suggest potential interactions between *PHT2* and proteins Calvin-Benson cycle-Related Binding protein (CRB), Glyceraldehyde-3-Phosphate Dehydrogenase A subunit 2 (GPA-2), Phosphoribulokinase (PRK), Zeatin-related Kinase Transporter (ZKT), and Proton Gradient Regulation 5 (PGR5). Notably, prediction scores for all potential protein interactions exceeded 0.9, indicating high confidence in the prediction outcomes.

### 2.3. Phylogenetic Analysis of PHT2 in Sweet Potato and Multiple Species: Interspecific Collinearity Analysis of PHT2 Gene

**Figure 3:**
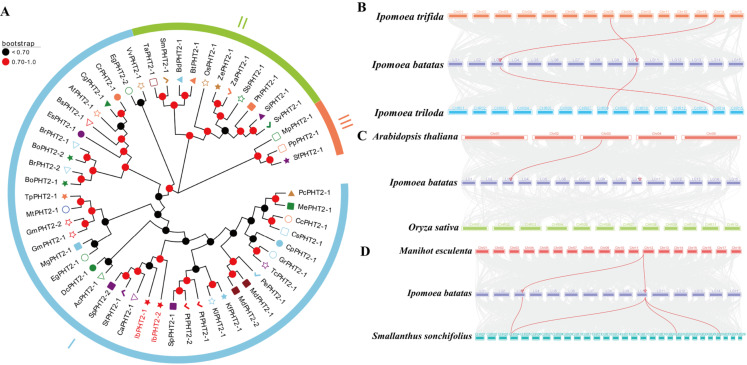
(**A**) Phylogenetic tree comprising *PHT2* family members from 46 species. *IbPHT2-1* (*g11922.t1*) and *IbPHT2-2* (*g40754.t1*) represent sweet potato. The tree includes diverse species such as *Ricinus communis* (*PcPHT2-1*), *Manihot esculenta* (*MePHT2-1*), *Citrus clementina* (*CcPHT2-1*), *Citrus sinensis* (*CsPHT2-1*), *Carica papaya* (*CpPHT2-1*), *Gossypium raimondii* (*GrPHT2-1*), *Theobroma cacao* (*TcPHT2-1*), *Prunus persica* (*PePHT2-1*), *Malus domestica Borkh* (*MdPHT2-1*, *MdPHT2-2*), *Kalanchoe fedtschenkoi* (*KfPHT2-1*), *Kalanchoe laxiflora* (*KlPHT2-1*), *Populus trichocarpa* (*PtPHT2-1*, *PtPHT2-2*), *Salix purpurea* (*SpPHT2-1*, *SpPHT2-2)*, *Solanum tuberosum* (*StPHT2-1*), *Aquilegia coerulea* (*AcPHT2-1*), *Daucus carota* (*DcPHT2-1*), *Eucalyptus grandis* (*EgPHT2-1*, *EgPHT2-2*), *Mimulus guttatus* (*MgPHT2-1*), *Glycine max* (*GmPHT2-1*, *GmPHT2-2*), *Medicago truncatula* (*MtPHT2-1*), *Trifolium pratense* (*TpPHT2-1*), *Brassica oleracea capitata* (*BoPHT2-1*, *BoPHT2-2*), *Brassica rapa FPsc* (*BrPHT2-1*, *BrPHT2-2*), *Eutrema salsugineum* (*EsPHT2-1*), *Boechera stricta* (*BsPHT2-1*), *Arabidopsis thaliana Columbia* (*AtPHT2-1*), *Capsella grandiflora* (*CgPHT2-1*), *Capsella rubella* (*CrPHT2-1*), *Vitis vinifera Genoscope.12X* (*VvPHT2-1*), *Triticum aestivum* L. (*TaPHT2-1*, *BdPHT2-1*), *Solanum melongena* L. (*SmPHT2-1*), *Oryza sativa* (*OsPHT2-1*), *Zea mays Ensembl-18* (*ZePHT2-1*), *Zea mays PH207* (*ZaPHT2-1*), *Sorghum bicolor* (*SbPHT2-1*), *Panicum hallii* (*PhPHT2-1*), *Setaria italica* (*SiPHT2-1*), *Setaria viridis* (*SvPHT2-1*), *Marchantia polymorpha* (*MpPHT2-1*), and *Sphagnum fallax* (*SfPHT2-1*). Black dots represent bootstrapping values < 0.7, and red dots represent bootstrapping values 0.7–1.0. Higher levels of bootstrap serve as an indicator for superior credibility of results. Different symbols in the figure represent specific species, where the red solid stars show the two members of sweet potato. *Arabidopsis* (**B**–**D**) Homology analysis of the *PHT2* gene across multiple species. The figures illustrate the comparative analysis of sweet potato with six other species: (**B**) *Ipomoea trifida* (triple-lobed petunia), *Ipomoea batatas* (sweet potato), and *Ipomoea triloba* (triple-lobed sweet potato). (**C**) *Arabidopsis thaliana* (*Arabidopsis*), *Ipomoea triloba* (sweet potato), and *Oryza sativa* (rice). (**D**) *Manihot esculenta* (cassava), *Ipomoea triloba* (sweet potato), and *Smallanthus sonchifolius* (yacon).

A systematic neighbor-joining (NJ) tree examined *PHT2* gene families between 46 species and the sweet potato *PHT2* family in order to understand their phylogenetic relationships. (Figure 3A). Figure 3A shows that sweet potato, potato, and pepper share a common evolutionary tree with red-skinned willow, which indicates an evolutionary connection between these plants. Phylogenetic trees assemble clustered genes based on functional similarities detected between genes, thus positioning them together as groups on their evolutionary tree [27]. The phylogenetic analysis shows two sweet potato genes grouped together, which indicates that their biological functions are highly similar.

To further investigate the sweet potato *PHT2* gene family, collinearity analysis was conducted on seven species: *Ipomoea batatas*, *Ipomoea trifida*, *Ipomoea triloba*, *Arabidopsis thaliana*, *Oryza sativa*, *Manihot esculenta*, and *Smallanthus sonchifolius* (Figure 3B–D).

The genomic organization of sweet potato resembled these taxa the most: *Ipomoea trifida*, *Ipomoea triloba*, and *Manihot esculenta*, along with *Smallanthus sonchifolius*, because the species exhibited closer genetic relatedness. The highest similarity between sweet potato and *Smallanthus sonchifolius* occurred because yacon possessed either better complete genome data or more accurate gene annotation in this region. The genetic connection between sweet potato and Ipomoea trifida and Ipomoea triloba stands out because their gene regions showed significant overlap, which shows the two species have the most closely related genomes.

### 2.4. Analysis of PHT2 Phosphorus Transporter Promoter, Phosphorylation Sites, and Trans-Membrane Structural Regions

**Figure 4:**
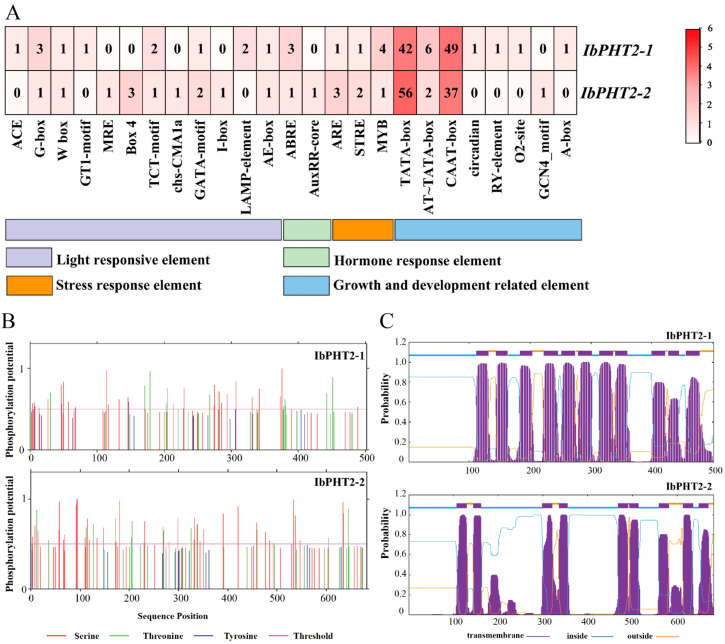
Functional prediction analysis of sweet potato *PHT2*. (**A**) Distribution of cis-acting elements in the sweet potato *IbPHT2* gene family. The intensity of red coloration corresponds to the number of homeopathic elements. (**B**) Prediction of *IbPHT2* phosphorylation sites in sweet potato. (**C**) Schematic representation of amino acid arrangement in the trans-membrane domain structure of sweet potato *PHT2* protein.

In order to illuminate the transcriptional control and prospective functional responsibilities of the *PHT2* gene, an examination of cis-regulatory elements within the promoter sequence was undertaken. Plant gene promoters play a crucial role in binding RNA polymerase and specifically activating the transcription of downstream structural genes, thereby controlling the direction and efficiency of transcription. Plants can respond to environmental changes through the regulation of gene transcription levels [28]. The Plant CARE website was utilized to predict the 2000 bp region upstream of the start codon for two members of the sweet potato *PHT2* family. The functional cis-regulatory elements in the promoters of these two members were sorted into four cohorts: light-responsive elements, hormone-responsive elements, stress-responsive elements, and growth- and development-related elements. Specifically, these include stress-responsive elements (ARE, STRE, MYB), various light-responsive elements (ACE, G-box, GT1-motif, etc.), abscisic acid-responsive element ABRE, auxin response regulator AuxRR-core, anaerobic induction regulator element ARE, and various growth- and development-related elements (TATA-box, CAAT-box, etc.). The predicted results underscore the significance of these elements in mitigating phosphorus deficiency stress (Figure 4A).

The study of protein phosphorylation sites can provide insights into their functions and regulatory mechanisms (Figure 4B). The figure illustrates the potential phosphorylation sites of *IbPHT2-1* and *IbPHT2-2* proteins, two members of the sweet potato *PHT2* family. Amino acids exceeding the threshold are generally considered more reliable. The *PHT2*-1 protein contained 29 serines, 12 threonines, and 4 tyrosines, while the *PHT2*-2 protein comprised 38 serines and 14 threonines. Although *IbPHT2-1* exhibited more phosphorylation sites compared to *IbPHT2-2*, serine phosphorylation sites predominated in both proteins, followed by threonine and tyrosine potential sites. Serine phosphorylation was prevalent in plant signal transduction and may regulate protein activity through phosphorylation or dephosphorylation switches.

The trans-membrane domain prediction results revealed 20 trans-membrane domains in sweet potato *PHT2* phosphorus transporters, with 11 in *IbPHT2-1* and 9 in *IbPHT2-2*. The N-terminal of the *IbPHT2-1* protein was located intracellularly (1-111aa), while its C-terminal was extracellular. Similarly, the N-terminal of the *IbPHT2-2* protein was intracellular (1-107aa), with its C-terminal was positioned extracellularly. The specific amino acid locations are provided in the attachment (Figure 4C, Tables S2 and S3).

### 2.5. Construction and Analysis of IbPHT2 Gene Expression Profile in Sweet Potato

**Figure 5:**
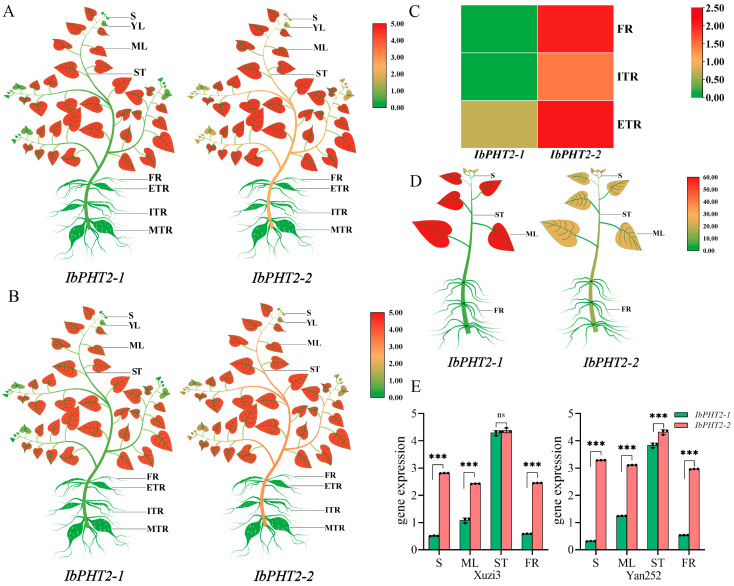
*PHT2* gene expression profile of Sweet potato. (**A**) Heat map representing tissue-specific expression in ‘Xuzi3’. S: shoot; ML: mature leaves; ST: stem; FR: fibrous root; ITR: initial tuberous root; ETR: expanding tuberous root; MTR: mature tuberous root. (**B**) Heat map depicting tissue-specific expression within ‘Yan252’, with abbreviations consistent with ‘Xuzi3’. (**C**) Heat map illustrating tissue-specific expression in ‘Annayu’. The FPKM values for the eight tissues of ‘Xuzi3’ and ‘Yan252’, as well as the three root types of ‘Annayu’, were log2 (FPKM+1) transformed to generate heat maps. (**D**) Heat map representing tissue-specific expression in “Heart Fragrance”. Expression values are presented as the average of three independent biological replicates, relative to root expression. Elevated expression levels are represented by red, whereas reduced expression levels are signified by green. (**E**) Tissue-specific expression of ‘Xuzi3’ and ‘Yan252’, The FPKM value is converted by log2(FPKM+1) to draw a histogram. Significance is denoted as: *** *p* < 0.001, ns: not significant.

*IbPHT2* gene expression levels were investigated using transcriptome data from ‘Xuzi3’ and ‘Yan252’ across eight distinct tissues (shoot, young leaf, mature leaf, stem, fibrous root, initial tuberous root, expanding tuberous root, and mature tuberous root). Generally, similar expression patterns were observed for the same gene between the two varieties (Figure 5A,B,E). In both ‘Xuzi3’ and ‘Yan252’, the *IbPHT2* gene exhibited the highest expression in mature leaves, followed by shoots, young leaves, and stems. Notably, *IbPHT2-2* demonstrated higher expression levels than *IbPHT2-1* in these four tissues, while no detectable expression of the *IbPHT2* gene was observed in the roots.

Interestingly, the ‘Annayu’ transcriptome data revealed a distinct expression pattern (Figure 5C). *IbPHT2-2* was highly expressed in fibrous roots, initial tubers, and mature tubers, whereas *IbPHT2-1* expression was not detected in fibrous roots and initial tubers. This divergent expression pattern suggests that *IbPHT2-2* may play a role in phosphorus absorption or distribution within the root system.

To validate the RNA-seq data, reverse transcription quantitative polymerase chain reaction (RT-qPCR) was performed to assess *IbPHT2* expression levels in ‘Xinxiang’ (Figure 5D,E). The results confirmed a tissue-specific transcriptional accumulation pattern for the *IbPHT2* gene. Both *IbPHT2-1* and *IbPHT2-2* were expressed in shoots, mature leaves, stems, and fibrous roots, with the highest expression levels observed in mature leaves, followed by shoots, stems, and fibrous roots. The preferential expression of *IbPHT2* genes in green tissues and fibrous roots suggests their potential involvement in the growth and development of green tissues and in phosphorus uptake or distribution within the root system.

### 2.6. Consequences of Phosphorus Deficiency Stress on the Development of Sweet Potato Seedlings

**Figure 6:**
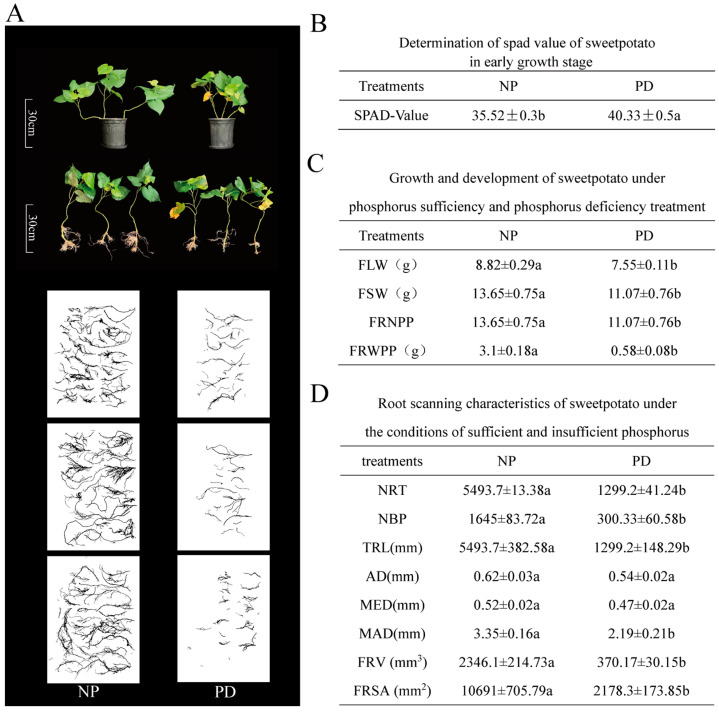
Phenotypic alterations under NP and PD treatments. (**A**) Impact of phosphorus deficiency on sweet potato seedling growth. (**B**) Determination of Spad values under normal and phosphorus deficiency treatments during the early growth stage of sweet potato. (**C**) One-way analysis of variance for sweet potato agronomic traits. LSD values are presented with standard deviations following the “+” symbol, and lowercase letters following each indicator denote remarkable disparities observed between the treatment groups (*p* < 0.05). The presented data represent the average of five replicate measurements (n = 5). FLW: fresh leaf weight; FSW: fresh stem weight; FRNPP: fibrous root number per plant; FRWPP: fiber root weight per plant. (**D**) Root scanning results for NP and PD treatments. NRT: number of root tips; NBP: number of branch points; TRL: total root length; AD: average diameter; MED: median diameter; MAD: maximum diameter; FRV: fiber root volume; FRSA: fiber root surface area.

The ‘Xinxiang’ variety was exposed to phosphorus deficiency for 15 days to study abiotic stress effects on sweet potato (Figure 6A). Phosphorus deficiency treatment (PD) significantly reduced leaf fresh weight, stem fresh weight, root fresh weight, and root number compared to normal phosphorus treatment (NP). Notably, fiber root weight exhibited an extremely significant decrease (Figure 6C). This observation suggests that phosphorus deficiency severely inhibits root growth and development, consequently impairing water and nutrient absorption, which in turn affects overall plant growth. Furthermore, chlorophyll content was measured in the fourth functional leaf of sweet potato. The results revealed a significant increase in chlorophyll content under phosphorus deficiency stress contrasted with normal treatment (Figure 6B). Root scanning data of sweet potato is presented in Figure 6D. Phosphorus deficiency significantly decreased several root scanning indicators, including number of root tips (NRT), number of branch points (NBP), total root length (TRL), maximum diameter (MAD), fiber root volume (FRV), and fiber root surface area (FRSA), when compared to normal treatment. However, average diameter (AD) and median diameter (MED) did not exhibit significant differences between NP and PD treatments. These findings collectively indicate that phosphorus deficiency impedes the growth and development of sweet potato, with a particularly pronounced effect on root development.

### 2.7. Analysis of Gene Expression Under Phosphorus Deficiency Stress

**Figure 7:**
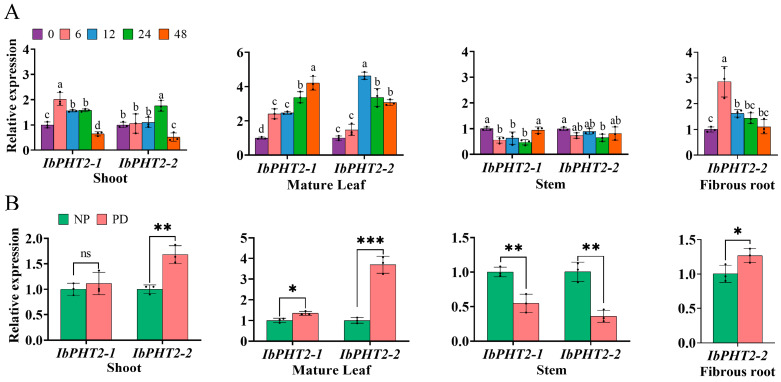
Temporal expression patterns and phenotypic alterations of *IbPHT2* in sweet potato cultivar ‘Xinxiang’ under phosphorus deficiency stress and normal conditions. (**A**) *IbPHT2* expression was analyzed over time under phosphorus deficiency. Plants were treated with phosphorus-free solution for 0, 3, 6, 12, 24, and 48 h. Expression levels are shown relative to the 0-h baseline. Analysis of variance was conducted with one-way analysis of variance, and least significant difference tests were applied. Treatment replicates were three, and different lowercase letters represent significant differences between treatments (*p* < 0.05). (**B**) *IbPHT2* expression was compared under normal phosphorus (NP) and phosphorus deficiency (PD) conditions. RT-qPCR was used to quantify expression in shoots, mature leaves, stems, and fibrous roots after 15 days. Each treatment had three replicates. Significance is denoted as: * *p* < 0.05, ** *p* < 0.01, *** *p* < 0.001, ns: not significant.

The tissue expression profiles of the four *IbPHT2* variants revealed high expression levels in shoots, mature leaves, and stems, with *IbPHT2-2* exclusively expressed in roots. RT-qPCR analysis was performed on shoots, mature leaves, stems, and fibrous roots to determine *IbPHT2* transcriptional dynamics under phosphorus deficiency stress. The results demonstrated temporal variations in *IbPHT2* expression across different tissues (Figure 7A). Significant disparities in *IbPHT2* gene expression were observed between shoots and mature leaves. After 6 h of phosphorus deprivation, *IbPHT2-1* expression in shoots peaked, exhibiting a 102% increase. Concurrently, *IbPHT2-2* expression in fibrous roots reached its maximum, with a 186% increase. At the 12-h mark, *IbPHT2-2* expression in mature leaves attained its zenith, showing a 363% increase. Following 24 h of stress, *IbPHT2-2* expression in shoots reached its apex, increasing by 75%. At 48 h, *IbPHT2-1* expression in mature leaves peaked, demonstrating a 320% increase. These findings indicate that under phosphorus deficiency stress, a preponderance of genes exhibited an initial upregulation in expression before subsequently declining. Conversely, *IbPHT2* gene expression in stems exhibited a continuous decrease over time. These observations suggest that *IbPHT2-1* and *IbPHT2-2* respond differentially to phosphorus deficiency stress and may serve distinct functions in various plant organs.

To further elucidate the role of *IbPHT2* in sweet potato growth and development, gene expression was assessed in four distinct tissues cultured under phosphorus deficiency stress for 15 days employing RT-PCR (Figure 7B). Under these conditions, *IbPHT2-1* expression in shoots was upregulated, albeit not significantly, while *IbPHT2-2* expression increased significantly. Both *IbPHT2-1* and *IbPHT2-2* were significantly upregulated in mature leaves, with *IbPHT2-2* exhibiting a highly significant difference. In contrast, stem tissue displayed decreased expression levels for both *IbPHT2-1* and *IbPHT2-2*, with significant differences observed. Root tissue showed a significant increase in *IbPHT2-2* expression. Notably, *IbPHT2-2* expression in mature leaves under phosphorus deficiency stress was highly significantly upregulated, increasing by 270%. In comparison to *IbPHT2-1*, *IbPHT2-2* appears to play a more pivotal role in responding to phosphorus stress, potentially aiding sweet potatoes in coping with phosphorus deficiency and optimizing Pi utilization.

### 2.8. Correlation Between IbPHT2 Gene Expression and Agronomic Traits

**Figure 8:**
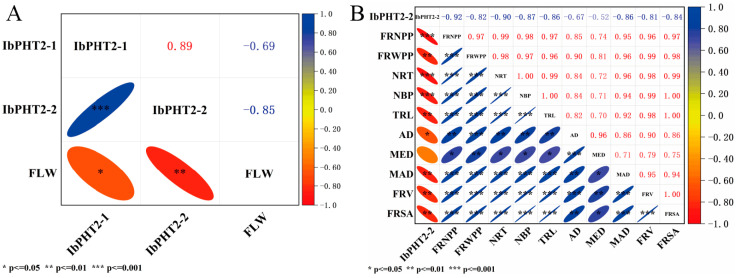
An investigation of the correlative relationships between *IbPHT2* gene expression levels as well as agronomic traits. (**A**) Correlation analysis between *IbPHT2* gene expression level and leaf fresh weight. (**B**) Correlation analysis of *IbPHT2-2* gene expression level with FRNPP, FRWPP, NRT, NBP, TRL, AD, MED, MAD, FRV and FRSA.

Pearson’s correlation coefficient analysis showed a strong negative correlation involving *IbPHT2* expression and various agronomic traits (Figure 8A), The results revealed a strong negative correlation between fresh leaf weight (FLW) and the expression of *IbPHT2-1* (r = −0.69) and *IbPHT2-2* (r = −0.85) in mature leaves. In fibrous roots (Figure 8B), *IbPHT2-2* expression exhibited significant negative correlations with multiple agronomic parameters, including the fibrous root number per plant (FRNPP; r = −0.92), fibrous root weight per plant (FRWPP; r = −0.82), number of root tips (NRT; r = −0.90), number of branching points (NBP; r = −0.87), total root length (TRL; r = −0.86), average diameter (AD; r = −0.67), maximum effective diameter (MED; r = −0.52), maximum actual diameter (MAD; r = −0.86), fibrous root volume (FRV; r = −0.81), and fibrous root surface area (FRSA; r = −0.84). All correlations, except for MED, reached statistical significance. These findings suggest that phosphorus deficiency impairs *IbPHT2* function, which positively affects the distribution and accumulation of inorganic Pi in mature leaves and fibrous roots [23,26]. Thus, this impairment prevents the maturation and development of sweet potato leaves and roots.

## 3. Discussion

### 3.1. Characteristic Analysis of PHT2 Gene Family

Sweet potato is a major crop in tropical and subtropical regions, serving as a primary source of food and industrial raw materials globally [29]. However, phosphate deficiency stress in acidic soils often impedes its growth and development [30]. This stress inhibits primary root growth while promoting lateral root development, thereby hindering water and nutrient absorption, affecting photosynthesis and overall plant growth, and ultimately leading to reduced crop yield [31]. The *PHT2* family plays a pivotal role in regulating chloroplast phosphate levels, being involved in trans-membrane transport, distribution, and phosphate homeostasis regulation [32], and is essential for plant response to phosphate deficiency stress. In this study, two members of the *PHT2* gene family were identified in sweet potato. Subcellular localization predictions indicated their presence in the chloroplast (Figure 1), corroborating previous findings on *Arabidopsis PHT2;1* [13]. Analysis of conserved motifs revealed that both *PHT2* genes contained motifs 1–10 (Figure 1B,C). This abundance of conserved motifs surpasses that found in *Arabidopsis*, cabbage, and pepper [33], and more closely aligns with findings in tomato, which contains motifs 1–8 [19]. The *PHT2* gene family possesses a single conserved domain, PHO4, consistent with studies in potato [18], suggesting its potential function as a Na(+)-phosphate symporter [34]. In the protein–protein interaction network prediction (Figure 2D), the *PHT2* protein exhibited the strongest link to the PRK protein. Previous research has postulated that PRK may enhance photosynthesis, providing the necessary energy to support the efficient operation of *PHT2;1*, thereby promoting phosphate accumulation [35]. A phylogenetic tree constructed employing 46 species revealed that the number of *PHT2* family members varied between one and two across species. Sweet potato was found to cluster with potato, pepper, and red-skinned willow in the same branch, possibly due to a shared evolutionary ancestor. Although these species belong to different families and genera, their clustering in the phylogenetic tree reflects functional similarity of their genes. Additionally, the two sweet potato genes were found to cluster together, consistent with findings in soybean [27], indicating functional similarity between the two sweet potato members (Figure 3A). Prediction of cis-acting elements revealed that both *IbPHT2-1* and *IbPHT2-2* contain stress response elements such as MYB transcription factors. These factors can specifically bind to the P1BS (PHR1-binding sequence) element, thereby activating the transcription of phosphate starvation-induced genes [36] and enhancing sweet potato’s resistance to phosphate deficiency stress. Furthermore, the *IbPHT2* gene contains multiple growth and development-related response elements as well as light response-related elements, indicating that the *PHT2* gene family plays a crucial regulatory role in plant light response and growth development processes (Figure 4A). In this study, *IbPHT2* was found to contain 11 and 9 trans-membrane regions, respectively, confirming its nature as a trans-membrane protein. This finding aligns with observations in potato, where the *StPHT2.1* gene contains 13 trans-membrane regions, and in *Arabidopsis*, which contains 12 trans-membrane regions [13,18] (Figure 4C). These findings collectively suggest that the *PHT2* gene family is highly conserved throughout plant evolution.

### 3.2. Impact of Phosphorus Deficiency Stress on Sweet Potato Seedling Growth

The study demonstrates that phosphorus deficiency stress significantly impaired various growth parameters of sweet potato seedlings, including leaf, stem, and root fresh weights, and the number of heels and root tips (Figure 6C). The root system’s growth and development were particularly affected by this stress. Visual manifestations included the chlorosis and yellowing of lower-positioned leaves, which eventually developed necrotic areas before aging and withering. Functional leaves exhibited a dark green coloration, while stems became thin and stunted. Additionally, the root system displayed poor development (Figure 6A). These observations align with previous research findings, which have indicated that phosphorus deficiency inhibits sweet potato root growth, reduces phosphorus utilization efficiency, and ultimately constrains overall plant growth [29]. Interestingly, the SPAD value of functional leaves in sweet potatoes subjected to phosphorus deficiency stress was significantly higher compared to those under normal phosphorus (NP) treatment (Figure 6B). This observation appears to contradict earlier studies, which have reported that phosphorus deficiency in plants leads to decreased chlorophyll content and subsequent inhibition of photosynthesis [37]. The discrepancy may be attributed to variations in stress resistance among different crop species. It is hypothesized that some plants, like sweet potatoes, may raise chlorophyll levels to offset photosynthesis decline from phosphorus deficiency [38].

### 3.3. Analysis of IbPHT2 Expression Patterns and Phosphorus Deficiency Stress Response

*IbPHT2* expression in ‘Xuzi3’ and ‘Yan252’ was highest in mature leaves, exceeding shoots, young leaves, and stems. Notably, *IbPHT2* expression was absent in roots. However, the expression profile in ‘Annayu’ cultivar diverges, with *IbPHT2-2* exhibiting high expression in fiber roots. To validate these transcriptome data, RT-qPCR analysis was conducted on ‘Xinxiang’ cultivar. The results corroborated the transcriptome data, demonstrating similar expression trends. *IbPHT2* was primarily expressed in shoots and mature leaves, while *IbPHT2-2* was expressed in fiber roots. A notable deviation from previous data was the higher expression of *IbPHT2-2* in shoots compared to mature leaves. To elucidate the transcriptional changes of *IbPHT2* under phosphorus deficiency stress, RT-qPCR analyzed *IbPHT2-1/2* expression in leaves, shoots, stems, and roots under phosphorus deficiency. The results revealed a significant upregulation of *IbPHT2-1*/2 in shoots and mature leaves under phosphorus deficiency conditions. Notably, *IbPHT2-2* expression in mature leaves substantially exceeded that of *IbPHT2-1*. Furthermore, *IbPHT2-2* expression in fiber roots increased significantly, suggesting a more crucial role for *IbPHT2-2* in phosphorus deficiency stress response. Conversely, *IbPHT2-1*/2 expression in stems decreased markedly. Based on these observations, we hypothesize that *IbPHT2-2* plays a more prominent role in shoots, mature leaves, and roots. This aligns with transcriptome results observed in Brassica species, where *PHT2* is significantly upregulated in shoots and mature leaves under phosphorus deficiency stress. However, our study diverges in that *PHT2* was also upregulated in stems [33]. In potato studies, *PHT2* expression in shoots remained unaltered, but significant upregulation was observed in leaves and stems, with comparable expression levels [18]. Root studies in poplar seedlings corroborate our findings, with *PtPHT2.2* significantly upregulated in roots under low phosphorus stress [21], suggesting a potential role in phosphorus absorption or distribution. The *PHT2* gene is localized in plastids. Rice research hypothesizes that root *PHT2* may indirectly influence phosphorus uptake by regulating phosphorus distribution in above-ground parts, thereby inhibiting leaf and root development [17]. Finally, *IbPHT2* gene expression levels in sweet potato were utilized to elucidate the response of fresh leaf weight (FLW), plant shoot weight (PSW), fibrous root number per plant (FRNPP), fibrous root weight per plant (FRWPP), and other indicators to phosphorus deficiency stress. Correlation showed *IbPHT2-2* expression negatively linked to agronomic traits, this limitation in gene function impedes the transporter’s ability to acquire sufficient phosphorus to meet plant requirements, ultimately leading to reduced performance of related agronomic traits in sweet potato.

## 4. Materials and Methods

### 4.1. Identification of Sweet Potato PHT2 Family

In this study, the sweet potato genome data and gene structure annotation files used were the ones available in the Ipomoea Genome Hub (sweetpotao.com/genome_jbrowse.html (accessed on 5 August 2023)) and the Sweet potato Genome Resources (http://sweetpotato.uga.edu/ (accessed on 7 August 2023)) [39]. *Arabidopsis* thaliana *PHT2* gene sequences were extracted from the *Arabidopsis* database (http://www.arabidopsis.org/, (accessed on 6 September 2023)). The Hidden Markov Model (HMM) of the *PHT2* conserved domain (PF01384) was acquired from the Pfam database (http://pfam.xfam.org/, (accessed on 7 September 2023)) [40]. The *Arabidopsis* protein sequence was utilized for sequence comparison via blast in TBtools v2.142, employing both HMM and blast methodologies to identify sweet potato *PHT2* genes. Gene structure and conserved domains were analyzed employing TBtools v2.142. To validate the accuracy of the *PHT2* gene family protein’s conserved structural domains, the NCBI CDD database (https://www.ncbi.nlm.nih.gov/Structure/bwrpsb/bwrpsb.cgi/ (accessed on 10 September 2023)) was employed, applying an E-value threshold of 1E-5 or lower. The definitive set of sweet potato *PHT2* family members was subsequently determined.

### 4.2. Physicochemical Properties and Subcellular Localization Prediction of Sweet Potato PHT2 Family Proteins

The ExPASy tool (https://www.expasy.org/ (accessed on 3 March 2024)) was employed to predict various physicochemical properties of the sweet potato *PHT2* family proteins, including amino acid length, isoelectric point, stability, and molecular weight. Subcellular localization was forecasted employing the online platform Cell-PLoc2.0 (http://www.csbio.sjtu.edu.cn/bioinf/Cell-PLoc-2/ (accessed on 4 March 2024)).

### 4.3. Chromosomal Location, Gene Structure, Conserved Motifs, and Domains Prediction of Sweet Potato PHT2 Genes

The MEME Suite (https://meme-suite.org/meme/doc/meme.html (accessed on 6 March 2024)) was utilized to visualize conserved motifs in the sweet potato *PHT2* protein sequences. Chromosomal mapping of *PHT2* genes and gene structure visualization were executed employing the GFF annotation files and CDS of the sweet potato genome in TBtools v2.142. Furthermore, TBtools v2.142 was employed to visualize conserved motifs and domains.

### 4.4. Secondary and Tertiary Structure Prediction and Protein Interaction Network of Sweet Potato PHT2 Proteins

The SOPMA web tool (NPS@ SOPMA secondary structure prediction—NPSA, Lyon, France (accessed on 12 March 2024)) was utilized to predict the secondary structure of sweet potato *PHT2* proteins. The tertiary structure of the *IbPHT2* protein was modeled based on homology employing the SWISS-MODEL tool (https://swissmodel.expasy.org/interactive, (accessed on 10 March 2024)). The protein–protein interaction network for sweet potato *PHT2* proteins was built employing the STRING database (STRING: functional protein association networks (accessed on 12 March 2024)), on the basis of homologous sequences from *Arabidopsis* thaliana.

### 4.5. Construction and Collinearity Assessment of the PHT2 Family Phylogenetic Tree in Sweet Potato

To ensure that we retrieved all *PHT2* protein sequences from various plants, we obtained the sequences from the PLAZA website (http://bioinformatics.psb.ugent.be/plaza/ (accessed on 15 March 2024)) (Table S4), and a phylogenetic tree of the *PHT2* gene family was constructed using the neighbor-joining (NJ) method in MEGA11 software [19]. The parameters were configured as follows: the substitution model was set to “p-distance”, gap treatment was designated as “Partial deletion”, and the bootstrap method value was established at 1000. Visualization of the phylogenetic tree was accomplished employing the online tool Evolview (http://www.evolgenius.info/evolview/#/ (accessed on 15 March 2024)). Interspecific collinearity analysis of the *PHT2* family across six species was rendered employing TBtools software.

### 4.6. Prediction of Cis-Acting Elements, Trans-Membrane Domains, and Phosphorylation Sites in the Sweet Potato PHT2 Protein Promoter

Putative cis-acting regulatory elements situated within the 2000 bp region upstream of the sweet potato *PHT2* gene promoter were identified through the utilization of the Plant CARE bioinformatic resource (https://bioinformatics.psb.ugent.be/webtools/plantcare/html (accessed on 20 March 2024)). Trans-membrane domains were forecasted through the TMHMM2.0 server (https://services.healthtech.dtu.dk/services/TMHMM-2.0/ (accessed on 21 March 2024)). Phosphorylation sites were identified utilizing the NetPhos 3.1 server (https://services.healthtech.dtu.dk/services/NetPhos-3.1/ (accessed on 22 March 2024)). 

### 4.7. Experimental Design

A pot experiment was conducted using the sweet potato variety “Xinxiang” as the experimental material at the Sanya Nanfan Research Institute of Hainan University (18°30′ N, 109°60′ E) in July 2024. The climatic conditions for the month were obtained from (https://www.qweather.com/ (accessed on 1 August 2024)). The total rainfall recorded was 66.2 mm, with a maximum temperature of 35 °C, a minimum temperature of 20 °C, and an average temperature of 26 °C. The experiment adopted a completely randomized design, with each treatment repeated 5 times, and 3 representative plants were taken each time, totaling 35 samples. The reason for the selection of the quartz sand was the particle size for the pot experiment: 0.2–0.4 mm, 0.4–0.6 mm, and 0.8–1.2 mm, and the ratio of the particle size was 1:2:1. The ultrapure water was used to thoroughly wash the mixed quartz sand three times. utilizing the sweet potato variety ‘Xinxiang’ as the experimental material. Virus-free seedlings exhibiting uniform growth tendencies were planted in pots (16 cm diameter, 17 cm height, 12 cm bottom diameter) employing an oblique insertion method. The seedlings were irrigated with standard Hoagland nutrient solution every three days. Ten days post planting, sweet potato seedlings demonstrating consistent growth were transferred to a phosphorus-deficient environment for cultivation. Sweet potato sprouts, mature leaves, stems, and fibrous roots were harvested at 0, 6, 12, 24, and 48 h post transfer, promptly submerged in liquid nitrogen and thereafter maintained at −80 °C. To simulate prolonged stress conditions, plants were administered Hoagland nutrient solution at three-day intervals. Sweet potatoes cultivated with normal Hoagland nutrient solution served as the control group, while those grown in phosphorus-deficient nutrient solution constituted the treatment group. After 15 days of cultivation, the agronomic traits of both the control and treatment groups were evaluated.

### 4.8. PHT2 Gene Transcriptome Data Download and RT-qPCR Analysis Under Phosphorus Deficiency Stress

Total RNA was extracted employing an RNA extraction kit (Tiangen, Beijing, China, DP441). One microgram of RNA was reverse transcribed into cDNA employing HiScript II Q RT SuperMix for RT-qPCR (Vazyme, Nanjing, China, R223). The expression of *IbPHT2* genes in sweet potato was analyzed employing RT-qPCR techniques. Gene-specific RT-qPCR primers were designed employing Primer Biosoft 5 kit (Primer Biosoft, Palo Alto, CA, USA) (Table 2). The actin housekeeping gene was employed as an internal control [41]. Quantitative RT-qPCR was performed with gene-specific primers using TB Green Premix Ex Taq II (Tli RNaseH Plus) (Takara, Tokyo, Japan, RR820A) on a RT-qPCR machine (qTOWER3G, Jena, Germany) for the RT-qPCR reaction. The thermal cycling protocol consisted of an initial denaturation step at 95 °C for 30 s, followed by 40 cycles of 95 °C for 15 s and 60 °C for 30 s. Relative expression levels were calculated employing the 2^−∆∆CT^ method, with the experiment comprising three independent biological replicates. The original transcriptome data for ‘Xuzi3’ and ‘Yan252’ were obtained from the official CNCB website (https://ngdc.cncb.ac.cn, (accessed on 2 August 2024)) under accession number CRA000606 [42]. ‘Annayu’ transcriptome data have been published in the GSA database (https://ngdc.cncb.ac.cn/gsa/, (accessed on 20 February 2025)), project number CRA023094.

### 4.9. Statistical Analysis

Statistical analyses were conducted employing analysis of variance (ANOVA) with Statistix 9 (Analytical Software, Tallahassee, FL, USA). GraphPad Prism 8.0.2.263 (GraphPad Software Inc., San Diego, CA, USA) was employed for generating bar charts. Correlation analyses were performed employing Origin 2024 (Origin Lab Corporation, Northampton, MA, USA). TBtools v2.142 [43] was utilized for graph creation and visualization.

## 5. Conclusions

The present investigation sought to identify and characterize the *PHT2* family within the sweet potato genome, comprising two members based on whole-genome analysis. Comprehensive analyses were conducted on the chromosomal locations, conserved motifs, gene structures, conserved domains, phylogenetic relationships, interspecific collinearity, cis-acting elements, phosphorylation sites, and trans-membrane domains of the sweet potato *PHT2* family. A pot experiment revealed that phosphorus deficiency stress significantly inhibited the progression as well as maturation of sweet potato leaves, stems, and fibrous roots. Tissue expression patterns of four sweet potato varieties, analyzed through transcriptome and RT-qPCR methods, demonstrated that *IbPHT2* was predominantly expressed in shoots, mature leaves, stems, and fibrous roots. Under phosphorus deficiency stress, *IbPHT2-2* expression was upregulated in shoots, mature leaves, and fibrous roots while being downregulated in stems. Correlation analysis indicated a negative correlation between *IbPHT2-2* expression in mature leaves and leaf fresh weight (FLW), as well as between its expression in roots and the root index. These findings indicated that phosphorus deficiency inhibited the function of *IbPHT2-2*, negatively impacting the distribution and accumulation of Pi in mature leaves and fibrous roots, thereby hindering the development of these tissues in sweet potato. This study provides preliminary evidence that *IbPHT2-2* is a pivotal genetic factor in the adaptive response to phosphorus deficiency stress, influencing Pi absorption as well as distribution in sweet potato. Furthermore, it establishes a foundation for future research on the molecular mechanisms underlying efficient Pi utilization in sweet potato.

## Figures and Tables

**Table 1:** Basic information of *PHT2* gene family members in sweet potato.

Gene Name	Gene ID	Protein Size (aa)	MW (kDa)	Isoelectric Point	Instability Index	GRAVY	Subcellular Localization
*IbPHT2-1*	g11922.t1	502	52.30	9.13	25.44	0.521	Chloroplast
*IbPHT2-2*	g40754.t1	682	72.98	9.52	35.26	0.305	Chloroplast

Note: MW, molecular weight; GRAVY, grand average of hydropathicity.

**Table 2:** The RT-qPCR primer sequences.

Gene Name	Forward Primer Sequence (5′–3′)	Reverse Primer Sequence (5′–3′)
*actin*	TATGGTTGGGATGGGACAGAA	CGGTAAGAAGGACAGGGTGCT
*IbPHT2-1*	GACAGAAACTACCCAGACCAAGAAC	ACCCAAACACGCCGTAAACTATC
*IbPHT2-2*	AATGATGTCTCCAACGCAATAGGC	ACAATCTGAGTCCCGCTTAGTCC

## Data Availability

Data are available upon request due to privacy and ethical restrictions.

## Supplementary Materials

The following supporting information can be downloaded at: https://www.mdpi.com/article/10.3390/ijms26062681/s1.
