# Supplementary material for: Genome-Wide Identification and Expression Assessment for the Phosphate Transporter 2 Gene Family Within Sweet Potato Under Phosphorus Deficiency Stress
Source: Int J Mol Sci. 2025 Mar 17;26(6):2681. doi: 10.3390/ijms26062681 (PMC11942005; doi:10.3390/ijms26062681)
Supplement: Supplementary file 1 [file ijms-26-02681-s001.zip › ijms-3518544-supplementary/Supplementary File(s)/Table S1- S4.pdf]

Genome-wide Identification and Expression Assessment for *PHT2* Gene Family within Sweetpotato Under Phosphorus Deficiency Stress

Table S1 Secondary structure prediction of *IbPHT2*protein

Gene name	Number of α-helix	Ratio (%)	Number of extended strand	Ratio (%)	Number of β-Corner	Ratio (%)	Number of random coil	Ratio (%)
IbPHT2-1	271	53.98	48	9.56	16	3.19	167	33.27
IbPHT2-2	287	42.08	94	13.78	24	3.52	277	40.62

Note: shows the secondary structure prediction diagram of IbPHT2 protein, which is composed of four parts: α-helix, extended strand, β-turn, random coil, and the Ratio of the latter cell of each part indicates the proportion of secondary structure.

Table S2 Prediction of *IbPHT2-1* protein transmembrane domain structure

Amino acid site	Initial amino acid	Terminating amino acid
inside	1	111
TMhelix	112	131
outside	132	143
TMhelix	144	163
inside	164	183
TMhelix	184	203
outside	204	222
TMhelix	223	245
inside	246	251
TMhelix	252	274
outside	275	278
TMhelix	279	301
inside	302	313
TMhelix	314	336
outside	337	339
TMhelix	340	359
inside	360	399
TMhelix	400	422
outside	423	426
TMhelix	427	444

inside	445	455
TMhelix	456	478
outside	479	502

Table S3 Prediction of *IbPHT2-2* protein transmembrane domain structure

Amino acid site	Initial amino acid	Terminating amino acid
inside	1	107
TMhelix	108	130
outside	131	144
TMhelix	145	162
inside	163	298
TMhelix	299	321
outside	322	335
TMhelix	336	355
inside	356	467
TMhelix	468	490
outside	491	493
TMhelix	494	513
inside	514	558
TMhelix	559	581
outside	582	612
TMhelix	613	635
inside	636	647
TMhelix	648	670
outside	671	682

Table S4 *PHT2* family genes in different species

Number	Organism	Gene Accession number
1	Ricinus communis	27504.m000647
2	Triticum aestivum L.	AF156696
3	Capsicum annuum L.	AF533081
4	Arabidopsis lyrata	AL5G15360.t1
5	Aquilegia coerulea	Aqcoe3G144600.1
6	Arabidopsis thaliana	AT3G26570.1
7	Solanum melongena L.	AY293827
8	Boechera stricta	Bostr.0556s0327.1
9	Brachypodium distachyon	Bradi3g47550.1
10	Brachypodium stacei	Brast04G151800.1
11	Capsella grandiflora	Cagra.3598s0004.1
12	Capsella rubella	Carubv10016883m
13	Citrus clementina	Ciclev10031076m
14	Daucus carota	DCAR_026513
15	Medicago	EF094558
16	Carica papaya	evm.model.supercontig_33.20
17	Gossypium raimondii	Gorai.005G178500.1
18	Zea mays Ensembl-18	GRMZM2G092780 T01
19	Vitis vinifera Genoscope.12X	GSVIVT01005753001
20	Kalanchoe fedtschenkoi	Kaladp0034s0076.1
21	Kalanchoe laxiflora	Kalax.0023s0132.1
22	Oryza sativa	LOC_Os02g38020.1
23	Manihot esculenta	Manes.12G023200.1
24	Marchantia polymorpha	Mapoly0138s0013.1
25	Medicago truncatula	Medtr8g069390.1
26	Mimulus guttatus	Migut.K00598.1
27	Citrus sinensis	orange1.1g012038m
28	Panicum hallii	Pahal.A02359.1
29	Solanum tuberosum	PGSC0003DMT400047869
30	Physcomitrella patens	Pp3c9 7350V3.1
31	Prunus persica	Prupe.1G244400.1
32	Salix purpurea	SapurV1A.0317s0260.1
33	Setaria italica	Seita.1G216200.1
34	Setaria viridis	Sevir.1G220000.1
35	Sphagnum fallax	Sphfalx0052s0101.1
36	Theobroma cacao	Thecc1EG010516t1
37	Eutrema salsugineum	Thhalv10003891m
38	Trifolium pratense	Tp57577_TGAC_v2_mR NA18371
39	Zea mays PH207	Zm00008a022208

40	Brassica oleracea capitata	Bol032193
41	Brassica oleracea capitata	Bol042818
42	Brassica rapa FPsc	Brara.F03266.1
43	Brassica rapa FPsc	Brara.I00256.1
44	Eucalyptus grandis	Eucgr.B03285.1
45	Eucalyptus grandis	Eucgr.H02499.1
46	Glycine max	Glyma.08G282100
47	Glycine max	Glyma.18G144100
48	Sorghum bicolor	Sobic.004G199900.1
49	Populus trichocarpa	Potri.008G186600.1
50	Populus trichocarpa	Potri.010G046300.1
